# Lamellar-Structured Al_2_O_3_-SiO_2_ Nanofibrous Aerogels with Favorable Compression Resilience for Efficient High-Temperature Thermal Insulation

**DOI:** 10.3390/molecules31111934

**Published:** 2026-06-03

**Authors:** Yuxin Ma, Mengjiao Zhang, Wenqiang Wang, Hanwen Zhang, Wenzhe Li, Xiangxiang Gu, Qiuxia Fu, Haoru Shan

**Affiliations:** 1 School of Textile and Clothing, Nantong University, Nantong 226019, China; 2315310003@stmail.ntu.edu.cn (Y.M.); 2315320005@stmail.ntu.edu.cn (M.Z.); wwq1710@163.com (W.W.); 2415310020@stmail.ntu.edu.cn (H.Z.); 2315310022@stmail.ntu.edu.cn (W.L.); 2415320003@stmail.ntu.edu.cn (X.G.); 2 National and Local Joint Engineering Research Center of Technical Fiber Composites for Safety and Health, Nantong University, Nantong 226019, China

**Keywords:** lamellar, Al_2_O_3_-SiO_2_ nanofibers, aerogels, high-temperature thermal insulation, compression resilience

## Abstract

Ceramic nanofiber-based materials have wide applicability in high-temperature management and protection. The transformation of conventional two-dimensional ceramic nanofibrous membranes into three-dimensional nanofiber-based bulks can effectively improve their thermal insulation performance and expand their range of applications. Herein, lamellar-structured Al_2_O_3_-SiO_2_ nanofibrous aerogels (LASO NFAs) with varying inorganic binder contents were prepared via a sequence of processes involving face-to-face stacking, impregnation, and calcination, using flexible Al_2_O_3_-SiO_2_ nanofibrous membranes (ASO NFMs) as building units and aluminum dihydrogen phosphate as an inorganic binder. Varying the inorganic binder content in the aerogel matrix enables effective control over the compressive properties and interlayer spacing of the resulting aerogels. Specifically, the optimized LASO-20 NFAs demonstrated relatively good compression resilience, with a plastic deformation of 22.1% after undergoing 500 compressive cycles at a compressive strain of 50%. Moreover, profiting from the high-temperature resistance of ASO NFMs and substantial air content present within nanofiber interlayers, the LASO-20 NFAs with a thickness of 20 mm could effectively insulate against surface temperatures of 1000 °C down to 224 °C. Moreover, LASO-20 NFAs exhibited a room-temperature thermal conductivity of approximately 0.043 W·m^−1^·K^−1^, illustrating a favorable high-temperature thermal insulation characteristic. Furthermore, the LASO-20 NFAs presented promising service performance in extreme environments, providing a novel perspective in the development of new types of ceramic aerogels.

## 1. Introduction

High-temperature thermal insulation materials play crucial roles in effectively minimizing the inefficient dissipation of thermal energy in the fields of industrial production, chemical metallurgy, high-temperature catalysis, building insulation, pipeline transportation, etc., which significantly contributes to energy conservation and enhances energy utilization efficiency [1,2,3]. Thus, attention has continuously been paid to the prominent topic of developing high-temperature thermal insulation materials. At present, ceramic materials occupy a dominant position among the various types of high-temperature insulation materials. As a lightweight refractory material, ceramic fiber-based insulation materials not only exhibit high strength and modulus but also possess exceptional properties such as resistance to elevated temperatures, low thermal conductivity, and resilience against chemical corrosion [4,5,6,7]. Consequently, they have emerged as a focal point for academic and industrial communities both domestically and internationally.

Among the ceramic fiber-based insulation materials, traditional two-dimensional micro/nanofiber membranes have been utilized widely in various high-temperature thermal protection fields on account of their light weight, low thermal conductivity, and good mechanical flexibility [8,9]. However, the limited thickness and relatively low porosity of the ceramic fiber-based membranous materials greatly restrict the further improvement of their thermal insulation application capabilities and scenario expansion. Traditional ceramic aerogel materials are characterized by their lightweight nature, controllable thickness, and low thermal conductivity, presenting significant potential for use in high-temperature thermal protection [10,11,12]. Nevertheless, conventional aerogels are typically formed through particle aggregation. Their internally discontinuous bead-chain-like structure renders them susceptible to structural collapse under substantial external stress, thereby constraining practical applications [13,14,15]. Additionally, traditional aerogels often experience particle fusion during high-temperature usage, resulting in decreased porosity and compromised thermal insulation performance that fails to meet the rigorous demands of high-temperature thermal protection applications [16,17,18]. Therefore, converting two-dimensional micro/nanofiber membranes into three-dimensional (3D) micro/nanofiber bulk materials with controllable thicknesses, enhanced porosity, and tortuous heat transfer pathways presents an effective solution to the mechanical deficiencies, structural instability issues, and inadequate high-temperature resistance associated with traditional ceramic aerogels.

Currently, various methods have been introduced to realize the construction of 3D ceramic nanofibrous aerogels, including direct spinning [16,19,20,21,22], template-based approaches [10,23,24], the vacuum filtration shaping method [13,25], vacuum freeze-drying [26,27,28], and the layer-by-layer stacking technique [29,30,31]. Ceramic nanofiber sponges produced through direct spinning demonstrate certain compressive and rebound properties. However, the necessity for specialized collectors to gather the ceramic nanofibrous materials usually results in challenges such as irregular fiber shapes, difficulties in controlling thickness, and inadequate inter-fiber bonding. The template-based method realizes the preparation of 3D ceramic nanofiber aerogels with controllable geometries by depositing ceramic components onto the surface of a regularly shaped 3D fibrous template and then removing the template. Nonetheless, the 3D structure is susceptible to collapse during the template removal process because of the strong acid/alkali or high-temperature calcination treatment. As for vacuum filtration molding method, ceramic fibers are mixed with a binder and solvent to form a uniformly fiber mixed dispersion. The dispersion is transferred to porous molds; precursor fiber blocks are obtained through vacuum filtrating and drying. Finally, porous ceramic fiber aerogels are obtained after treating the precursor fiber blocks with high-temperature calcining. These methods have been demonstrated to be effective routes for the construction of ceramic fiber-based aerogels, yet the resultant ceramic aerogels generally suffer from unsatisfactory thickness tunability and comparatively large bulk density. The vacuum freeze-drying approach combines the homogeneous dispersion of the fiber building blocks and binder, low-temperature freezing, vacuum drying, and high-temperature calcination to prepare ceramic fiber aerogels. Generally, this technology can accurately control the dimension, shape and bulk density of ceramic aerogel materials. However, the internal ceramic short fibers of aerogels transfer external stress through point-to-point contact. When subjected to external force or exposed to a high temperature for a long time, the as-prepared ceramic aerogels are prone to structural collapse and strength degradation, which significantly reduces their stability and makes it difficult for them to withstand strong mechanical force or thermal shock in practical applications.

Different from the above-mentioned methods, the layered stacking approach constructs ceramic nanofiber aerogels by directly stacking ceramic fibrous membranes into 3D monolithic materials, followed by interlaminar bonding, drying, and subsequent high-temperature calcination. The laminated stacking method is simple and efficient, and the resulting ceramic nanofibrous aerogels exhibit an obvious multi-layered structure, which can effectively improve both compressive strength and structural stability. In addition, the porous characteristics both among the ceramic nanofibers and between the stacked membranous layers serve to retain the static air inside the aerogel, thereby reducing the temperature increase related to solid heat conduction. To date, several types of ceramic fiber aerogels have been prepared via the interlayer stacking method. For instance, Zhang et al. [30] developed ZrO_2_-SiO_2_ nanofibrous aerogels possessing a porous layered arch-shaped honeycomb architecture by processing the pre-shaped flexible ZrO_2_-SiO_2_ nanofiber membranes through silica sol soaking, ultrasonic treatment, stacking, freeze-drying, and calcination. The resultant ZrO_2_-SiO_2_ nanofiber aerogel presented excellent mechanical properties and low thermal conductivity. Although considerable advances have been made in the fabrication of ceramic nanofiber thermal insulators via the laminated stacking method, the types of ceramic nanofibers need to be further expanded. With prominent thermal stability, superior thermal shock endurance and outstanding chemical resistance, alumina-based fibrous materials are highly promising for high-temperature insulating applications. However, the traditional 3D alumina-based bulk fiber insulation materials (such as alumina-based aerogels) are hindered by their inherent brittleness and inadequate high-temperature performance, which severely restricts their practical applications.

Herein, flexible Al_2_O_3_-SiO_2_ nanofibrous membranes (ASO NFMs) were fabricated by integrating the sol–gel method with electrospinning technology. After that, lamellar-structured Al_2_O_3_-SiO_2_ nanofibrous aerogels (LASO NFAs) were prepared by taking electrospun ASO NFMs as structural units and aluminum dihydrogen phosphate as an inorganic adhesive through a 3D construction process involving stacking, impregnation and calcination. Benefitting from the favorable mechanical characteristics of the ASO NFMs, together with the laminated structure and effective face-to-face bonding networks, the resultant LASO NFAs demonstrated a desirable compression recovery. The optimized LASO-20 NFAs demonstrated comparatively stable compression performance even under liquid nitrogen and butane spray gun flame conditions. In addition, the high-temperature resistance of the ASO NFMs and the large amount of air contained within the nanofibrous membrane interlayers endowed the resultant aerogel with good thermal insulation properties. Moreover, the resultant aerogel exhibited good thermal protection and superior high-temperature service performance under extreme conditions, providing a novel perspective in the development of new types of structural ceramic aerogel materials.

## 2. Results and Discussion

**Figure 1:**
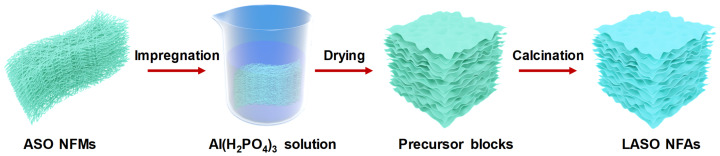
Schematic diagram of the preparation process for LASO NFAs.

The fabrication process for layered stacked Al_2_O_3_-SiO_2_ nanofiber bulks is shown in Figure 1. Typically, the electrospun ASO NFMs were stacked layer by layer and then impregnated into aluminum dihydrogen phosphate (Al(H_2_PO_4_)_3_) aqueous solutions. After immersing for 1 h, the wet blocks were removed from the Al(H_2_PO_4_)_3_ solution and then dried to obtain precursor blocks. Subsequently, the Al(H_2_PO_4_)_3_ within the uncrosslinked bulk was converted to AlPO_4_, promoting interlayer bonding between the membranes. Ultimately, the lamellar-structured LASO NFAs were obtained.

**Figure 2:**
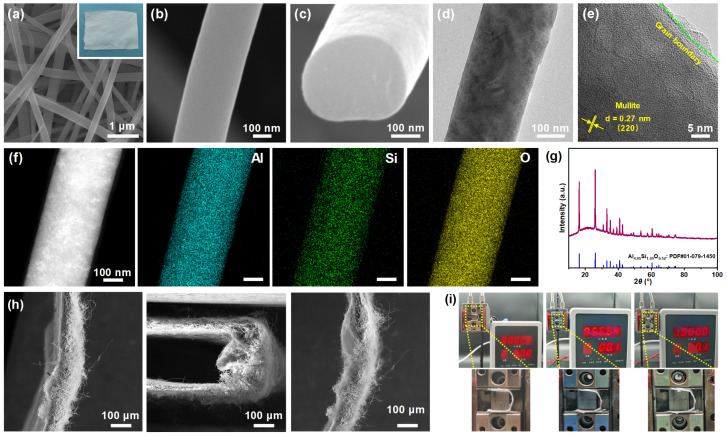
(**a**–**c**) SEM images and optical photographs (inset) of the as-spun ASO NFMs. (**d**,**e**) TEM images and (**f**) elemental distribution mapping of the single ASO nanofibers. (**g**) XRD pattern. (**h**) SEM images depicting the in situ bending process. (**i**) Optical photographs taken before and after 10,000 bending cycles of the ASO NFMs.

Since the lamellar-structured nanofibrous aerogels were formed by the electrospun ASO NFMs, the investigation of the microstructural characteristics, crystal structure, and mechanical performance of the ASO NFMs is necessary before the systematic research of the LASO NFAs. As can be seen from the FE-SEM images and the optical photograph (inset) of the resultant ASO NFMs presented in Figure 2a–c, the Al_2_O_3_-SiO_2_ nanofibers exhibited good continuity with a relatively uniform diameter. The single Al_2_O_3_-SiO_2_ nanofiber showed a relatively smooth single-fiber surface and well-rounded cross-sections. Further investigation into the microstructure of the ASO NFMs was conducted by TEM. As depicted in Figure 2d,e, the Al_2_O_3_-SiO_2_ nanofibers displayed a compact structure devoid of any noticeable macroscopic cracks. Both crystalline and amorphous regions coexist in the fibers, where the interplanar spacing of 0.27 nm corresponds to the (220) crystal plane of mullite.

In addition, as shown in Figure 2f, a uniform distribution of Al, Si, and O elements throughout the nanofiber could be observed from the energy-dispersive spectroscopy (EDS) mapping images. This observation indicated that a composite oxide consisting of Al and Si was formed from precursor fibers after calcination in an air atmosphere. Moreover, Al, Si, and O elements were evenly distributed on the single fiber. In addition, as shown in Figure 2g, distinct and intense crystallization peaks around 16.1°, 25.8°, 30.7°, 32.8°, 34.9°, 36.7°, 38.8°, 40.4°, 42.3°, 48.9°, 53.3°, 57.4°, and 60.3° further confirm the formation of a mullite phase (JCPDS PDF#01-079-1450) belonging to an orthorhombic crystal system.

Tensile and flexural tests were performed to investigate the mechanical properties of the ASO NFMs. As illustrated in Figure S1, the tensile strength of the ASO NFMs reached 1.1 MPa, accompanied by a flexural stiffness of 130 mN. After undergoing 500 flexural cycles, the flexural stiffness could stabilize at 61 mN (Figure S2), indicating that the fabricated ASO NFMs possess good tensile strength and flexibility. The bending-recovery evolution process of the ASO NFMs was examined using SEM. As depicted in Figure 2h, under applied stress, the fibers exhibited bending behavior and subsequently rebound upon removal of stress, showcasing their favorable flexibility. During the bending process of the fibrous membrane, inter-fiber sliding and the bending deformation of individual fibers can be clearly observed, which effectively relieves the internal bending stress. Furthermore, after undergoing 10,000 consecutive bending cycles, the ASO NFMs could still maintain desirable flexibility without any observable cracks (Figure 2i).

**Figure 3:**
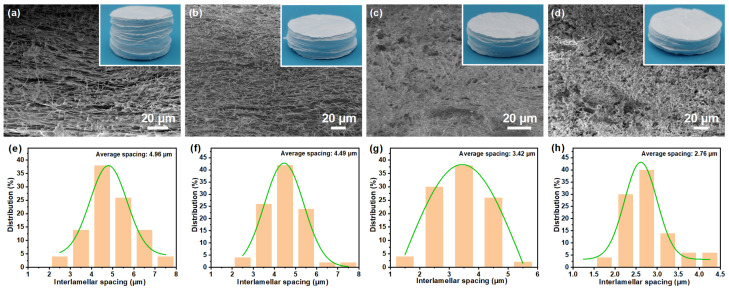
SEM images of the cross-section of LASO NFAs fabricated from different Al(H_2_PO_4_)_3_ loading amounts in aqueous solutions of (**a**) 5 wt%, (**b**) 10 wt%, (**c**) 20 wt%, and (**d**) 30 wt%. Insets show the optical photo of the corresponding samples. (**e**–**h**) Distribution histogram of the corresponding interlayer spacing between Al_2_O_3_-SiO_2_ nanofiber membranes within these LASO NFAs.

Since LASO NFAs were prepared via the face-to-face stacking of ceramic nanofibrous membranes, followed by impregnation in Al(H_2_PO_4_)_3_ solution, drying and high-temperature calcination, the material exhibits a unique stacked structure. Thus, the content of Al(H_2_PO_4_)_3_ in the immersion solution is an important factor affecting the interfacial adhesion between the fiber membranes. Accordingly, the microstructure structure of the LASO NFAs, fabricated from different Al(H_2_PO_4_)_3_ loading amounts, was systematically investigated. Figure 3a–d present the SEM images of the cross section and the corresponding optical photographs of the relevant LASO NFAs. Significantly, it can be seen from the optical images that with the increase in Al(H_2_PO_4_)_3_ loading amount, the packing density of the as-prepared LASO NFAs gradually increased and the height gradually decreased. As exhibited in the SEM images, the Al_2_O_3_-SiO_2_ nanofibrous blocks are distributed in layers. A certain gap filled with air can be observed between the layered stacked ASO NFMs, which is consistent with the design philosophy. In addition, with the increase in Al(H_2_PO_4_)_3_ content, the interlayer gap gradually fades, and the nanofiber bulk structure begins to evolve into a whole.

Furthermore, the interlayer spacing of the ASO NFMs was measured and the distribution histogram was drawn. As can be seen in Figure 3e–h, the LASO-5 and LASO-10 NFAs exhibited relatively dispersed interlayer distance distributions mainly in the range of 2 to 8 μm. The calculated average interlayer spacing of the LASO-5 and LASO-10 NFAs was 4.96 to 4.49 μm, respectively. The relatively large interlayer spacing size indicated that the interlayer adhesion of ASO NFMs within the aerogels is relatively weak at low Al(H_2_PO_4_)_3_ contents. As the loading amount of Al(H_2_PO_4_)_3_ increased to 20 wt%, the average interlayer spacing of the LASO NFAs decreased to 3.42 μm, and the distribution of the spacing changed from dispersion to concentration, indicating that interlayer adhesion had improved. As the content of Al(H_2_PO_4_)_3_ increased to 30 wt%, the LASO NFAs presented a smaller average interlayer spacing of 2.76 μm and more concentrated spacing size distribution. However, excessive Al(H_2_PO_4_)_3_ loading will lead to enhanced densification of aerogel materials, thus presenting brittleness and a lack of flexibility.

**Figure 4:**
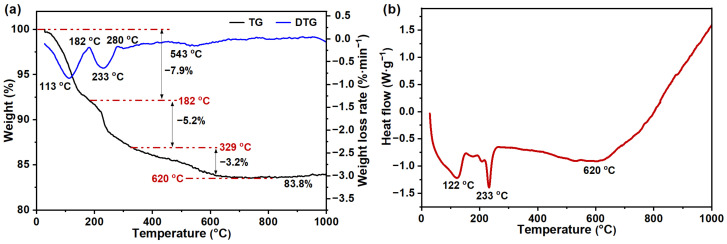
(**a**) TG-DTG and (**b**) DSC curves of the stacked ASO NFMs bulk after dipping with Al(H_2_PO_4_)_3_ and drying.

To investigate the crosslinking behavior of the uncrosslinked nanofibrous bulk materials at elevated temperatures, TG-DSC characterization was conducted on the stacked ASO NFMs bulk after dipping with Al(H_2_PO_4_)_3_ solution and drying. As illustrated in Figure 4a, the weight loss of the ceramic nanofibrous bulk from room temperature to 1000 °C can be categorized into three primary segments. The first segment, spanning from room temperature to 182 °C, exhibited a weight loss of approximately 7.9%. This stage primarily involved the removal of both physical and crystalline water, as well as the dehydration of Al(H_2_PO_4_)_3_ to form polyaluminum phosphate, which corresponds to the exothermic peak observed at 122 °C in the DSC curve shown in Figure 4b [32]. As for the second stage, from 182 °C to 329 °C, there is an additional weight loss of approximately 5.2%. This phase entailed the further decomposition of polyaluminum phosphate into pyrophosphoric acid aluminum and metaphosphoric acid aluminum, accompanied by the release of phosphoric acid gas, which contributes to weight reduction [33]. This process correlates with the exothermic peak at 233 °C in the DSC curve. Within the temperature range of 329 °C to 620 °C, a weight loss of about 3.2% occurred. This was predominantly due to further conversion processes involving the conversion of intermediate products such as aluminum metaphosphate into stable aluminum phosphate while releasing gaseous phosphorus pentoxide, which is reflected in the exothermic peak at 620 °C in the DSC curve. Moreover, a slight increase in weight is noted between temperatures of 620 °C and 1000 °C, which might be attributed to the PO-H groups present within the heated aluminum metaphosphate structures [34]. These hydroxyl groups decompose into H_2_O at around 875 °C; thus, a slight increase in sample weight can be observed.

**Figure 5:**
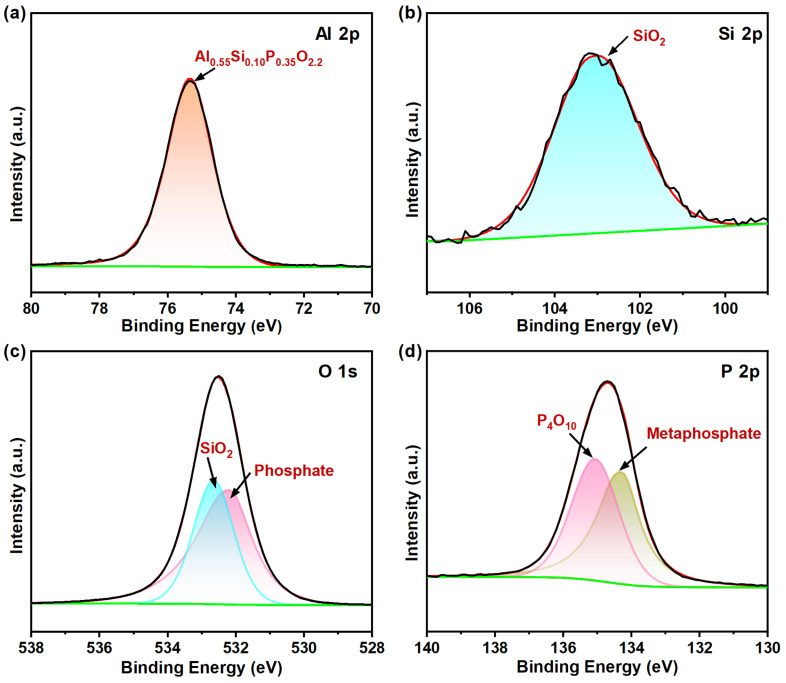
Deconvoluted high-resolution XPS spectra: (**a**) Al 2p, (**b**) Si 2p, (**c**) O 1s, and (**d**) P 2p in LASO-20 NFAs.

The full X-ray photoelectron spectroscopy (XPS) spectrum of the resultant LASO-20 NFAs (Figure S3) could reveal the presence of oxygen (O), silicon (Si), aluminum (Al), and phosphorus (P) elements on the aerogel samples. This indicates the successful loading of aluminum dihydrogen phosphate onto the fibers. Figure 5a displays the Al 2p XPS spectrum, highlighting a characteristic peak at 75.3 eV within the nanofibrous aerogels, primarily attributed to the Al_0.55_Si_0.1_P_0.35_O_2.2_. Figure 5b illustrates the XPS spectrum for Si 2p, showing a distinct peak at 103.0 eV for the nanofiber bulk, predominantly corresponding to the SiO_2_ component. Figure 5c presents the O 1s XPS spectrum, which shows two significant peaks at 532.2 eV and 532.7 eV for the fiber bulk material, mainly corresponding to metaphosphate and SiO_2_. Figure 5d depicts the XPS spectrum for P 2p, revealing two notable peaks at 134.3 eV and 135.1 eV in the fiber bulk, primarily associated with metaphosphate and P_4_O_10_.

**Figure 6:**
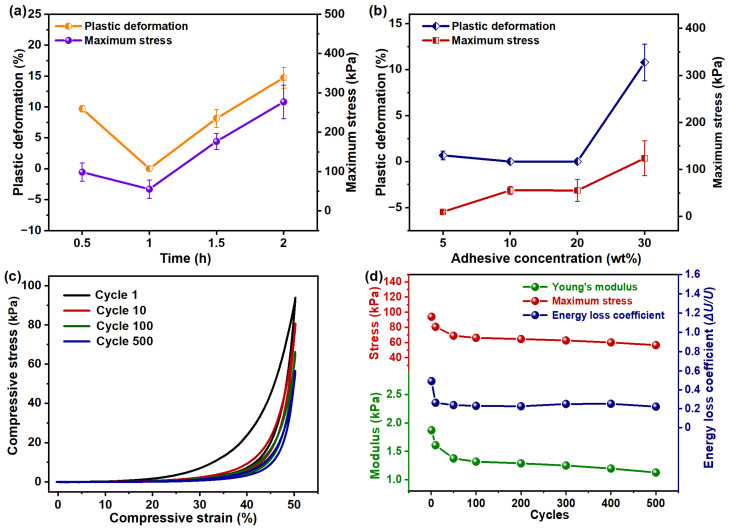
Plastic deformation and maximum stress of LASO NFAs: (**a**) different immersion times (fixed Al(H_2_PO_4_)_3_ concentration: 20 wt%); (**b**) different sol concentrations (fixed immersion time: 1 h); (**c**) representative stress–strain curves of the LASO-20 NFAs within 500 cycles of compression at 50% strain; (**d**) the corresponding variation trends of Young’s modulus, the energy loss coefficient, and the maximum stress of the LASO-20 NFAs in the cyclic compression process.

The compressive recovery performance of the ceramic nanofibrous aerogel is essential for ensuring their stable application during actual service as it directly influences the thermal insulation efficiency, service life, and environmental adaptability. Consequently, the compressive properties of the as-prepared LASO NFAs were systematically measured and investigated. The effects of impregnation time and binder concentration on both the plastic deformation and maximum compressive stress of the LASO NFAs (taking LASO-20 NFAs as an example) were examined under a fixed maximum strain of 50%. Figure 6a illustrates the compression mechanic properties of the corresponding LASO-20 NFAs obtained from various impregnation times (0.5, 1, 1.5, and 2 h). It is obvious that, as the impregnation time was set to 1 h, the resultant LASO-20 NFAs presented minimum stress (55.3 kPa) in the first compression cycle and almost no plastic deformation after 10 compressive cycles. This phenomenon can be attributed to the fact that the short impregnation time (0.5 h) caused the Al(H_2_PO_4_)_3_ in the solution to be unable to fully diffuse into the interior of the fiber block such that a stable fiber network structure could not be formed inside the aerogel after calcination. Consequently, localized collapse under external stress led to significant plastic deformation. As impregnation time increased, cross-linked networks gradually formed among Al_2_O_3_-SiO_2_ nanofibers within the LASO-20 NFAs, thereby significantly reducing its plastic deformation. However, a further increase in impregnation time would result in overly dense bonding points among nanofibers, which would greatly limit the bending and relative slippage of the nanofibers, thereby making it difficult for the LASO-20 NFAs to recover effectively after compression, thus showing larger compressive stress and plastic deformation.

Subsequently, the impact of Al(H_2_PO_4_)_3_ concentration on the compressive properties of the LASO NFAs was investigated. Figure 6b demonstrates that as the Al(H_2_PO_4_)_3_ loading amount increased from 5 wt% to 20 wt%, the plastic deformation decreased from 0.5% to nearly 0, and the maximum stress increased from 9.7 kPa to 55.3 kPa. This trend can primarily be attributed to the enhanced robustness of the inter-fiber crosslinking network resulting from increased Al(H_2_PO_4_)_3_ content, which enabled it to better withstand external stress and exhibit superior rebound properties. However, as the Al(H_2_PO_4_)_3_ concentration further increased to 30 wt%, the plastic deformation of the LASO-30 NFAs increased significantly to 10.8%, accompanied by an increase in maximum stress to 123.7 kPa. This phenomenon may be explained by excessive crosslinking points between the Al_2_O_3_-SiO_2_ nanofibers formed from the higher loading levels of Al(H_2_PO_4_)_3_. Consequently, stress dissipation of the LASO NFAs was hindered under external compressive force, leading to diminished rebound performance and an increase in compressive stress.

Based on the above results, LASO-20 NFAs (20 wt% Al(H_2_PO_4_)_3_ concentration, 1 h immersion time) were selected as the typical sample for subsequent compressive fatigue resistance characterization. As illustrated in Figure 6c, after undergoing 500 compressive cycles at a compressive strain of 50%, the LASO-20 NFAs exhibited a plastic deformation of 22.1%. Moreover, as presented in Table S1, compared with the reported lamellar ceramic fiber aerogels, the resultant LASO-20 NFAs demonstrated comparatively improved compression performance. A quantitative analysis of Young’s modulus, energy loss coefficient, and maximum stress across different numbers of compressive cycles was conducted. As presented in Figure 6d, both Young’s modulus and maximum stress decreased during the initial ten cycles before gradually stabilizing throughout the subsequent compression cycles. After completing 500 compression cycles, the peak stress and Young’s modulus of the LASO-20 fiber bulk retained over 60% of their initial values, while the energy loss coefficient reached 0.2, signifying relatively good flexural fatigue resistance for the resultant lamellar-structured ceramic nanofibrous aerogels.

**Figure 7:**
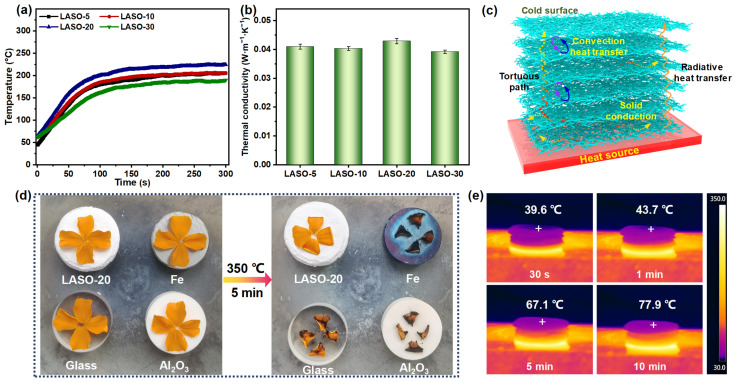
(**a**) Hot-plate test curves of the LASO NFAs at a hot-plate temperature of 1000 °C. (**b**) Thermal conductivity of the LASO NFAs at room temperature. (**c**) Schematic diagram of heat transfer pathway inside the LASO NFAs. (**d**) Thermal insulation capability of the LASO-20 NFAs compared to iron, glass, and Al_2_O_3_ ceramic in protecting fresh petals from wilting. (**e**) Infrared image of the LASO-20 NFAs within 10 min heating on a 350 °C hot plate.

The binder content is a critical factor influencing the interlayer spacing of nanofiber bulks, and this interlayer distance is closely associated with the air content within these aerogel materials. Consequently, the thermal insulation properties of nanofiber bulks (thickness of 20 mm) with varying binder contents were investigated. First, the thermal insulation performance of the resultant LASO NFAs at elevated temperatures was assessed using a high-temperature heating platform. As illustrated in Figure 7a, when these aerogels were placed on a hot stage with a hot-face temperature of 1000 °C, their surface temperature rose gradually and finally reached a stable state. When the loading amount of Al(H_2_PO_4_)_3_ was 5 wt% and 10 wt%, the corresponding LASO-5 and LASO-10 NFAs exhibited almost similar cold-side temperatures of around 205 °C, illustrating comparable thermal insulation capacity. As the Al(H_2_PO_4_)_3_ content increased to 20 wt%, the LASO-20 NFAs displayed a larger cold-side temperature of approximate 224 °C, which indicated a weakened thermal insulation property. By contrast, LASO-30 NFAs showed a decreased cold-side temperature of around 190 °C with improved high-temperature thermal insulation performance. Subsequently, the room-temperature thermal conductivity of LASO NFAs was measured via the hot-wire method. As displayed in Figure 7b, the thermal conductivity presents a trend of first slightly decreasing, then increasing, and finally decreasing, with the maximum value obtained at LASO-20 NFAs. The thermal conductivity values could be maintained between 0.039 and 0.043 W·m^−1^·K^−1^, showing favorable thermal insulation performance compared with previously reported materials (listed in Table S1).

This distinct variation tendency is presumed to be closely associated with the evolution of the internal layered structure. With increasing binder content, the interlayer spacing of fabricated aerogels gradually decreases, which might alter the dominant internal heat transfer forms. At a low binder dosage, large interlayer spacing can retain plenty of static air, and heat transfer is likely to be dominated by gas conduction and thermal convection. The moderate introduction of a binder may optimize the fiber stacking state and restrain convective and radiative heat transportation, which could contribute to the decline of thermal conductivity. When the binder content approaches the medium level of LASO-20, it is inferred that interconnected solid heat conduction channels are gradually established within the fibrous network. In this case, solid conduction, gas conduction and thermal convection proceed simultaneously, which probably accelerates the overall heat transfer and results in elevated thermal conductivity. Further increasing binder content is expected to further narrow interlayer gaps and largely restrict interlayer thermal convection. The overall heat transfer process may be dominated by solid-phase conduction and weak gas conduction, which may improve the thermal insulation property to a certain extent. Similar competitive heat transfer characteristics have been discussed in relevant research on fibrous thermal insulation materials. Such competitive heat transfer behavior in layered fibrous materials has also been reported in previous related studies [17,31]. The main heat transfer pathways inside LASO NFAs are illustrated in Figure 7c, including solid conduction along interconnected nanofiber networks, interlayer thermal convection, and interlayer thermal radiation. The tortuous fibrous structure prolongs the actual heat transfer path and hinders rapid heat propagation, which is conducive to achieving satisfactory thermal insulation performance.

In order to intuitively investigate the thermal insulation performance of the as-prepared ceramic aerogels, we placed the thermal insulation plates made of LASO-20 fiber blocks, iron, glass and Al_2_O_3_ ceramic on a hot stage at a temperature of 350 °C. After the fresh petals were placed on the top surface of each plate synchronously, the appearance changes in these corresponding petals were observed. As shown in Figure 7d, after 5 min of heating, only slight wilting could be observed from the petals placed on the LASO-20 NFAs, while those on other plates appeared visibly scorched and even carbonized, indicating the good thermal insulation performance of LASO-20 NFAs. Moreover, to further assess the thermal insulation performance of the LASO-20 NFAs, the dynamic temperature distribution of the surface of the aerogel placed on the hot plate was recorded using an infrared thermal imaging camera. As illustrated in Figure 7e, the temperature of the upper surface of the LASO-20 NFAs rose from a normal temperature to 40 °C after being placed on the hot plate (350 °C) for 30 s, and the surface temperature rose to 67 °C after 5 min. Surprisingly, the LASO-20 NFAs exhibited a relatively mild surface temperature of 78 °C even after 10 min, further indicating that the resultant lamellar-structured ceramic aerogels possess favorable thermal insulation performance.

**Figure 8:**
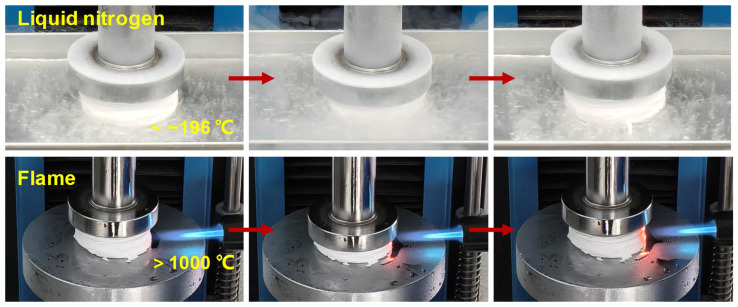
Optical photographs recording the compression and recovery processes of the LASO-20 NFAs in liquid nitrogen and butane torch flames.

To further evaluate the extreme temperature range of the resultant ceramic aerogels, the LASO-20 NFAs were selected as the representative samples for in situ compression tests in ultra-low-temperature liquid nitrogen (~−196 °C) and ultra-high-temperature butane spray gun flame (>1000 °C). As illustrated in Figure 8 and Figure S4, the LASO-20 NFAs exhibited a relatively good compressive strength, even when subjected to cryogenic liquid nitrogen and butane torch flames, without exhibiting any signs of cracking or combustion. Moreover, to directly verify the structural stability of the fabricated LASO-20 NFAs under high-temperature conditions, the fabricated aerogel was continuously calcined at 1300 °C for 24 h in a high-temperature furnace. As shown in Figure S5, the crystal phase could still remain stable compared with Figure 2g. Additionally, the macroscopic structural evolution of LASO-20 NFAs during continuous heating up to 1300 °C was monitored using an image-type sintering instrument (SJY, Xiangtan Xiangyi Instrument Co., Ltd., Xiangtan, Hunan, China). As indicated in Figure S6, during heating from 40 to 1000 °C at 10 °C·min^−1^, the bulk aerogel maintained a complete macroscopic structure without obvious delamination, cracking, or pulverization. When the temperature further increased to 1300 °C (with a reduced heating rate of 5 °C·min^−1^) and was maintained for 6 h, the aerogel exhibited a gradual shrinkage, but the overall monolithic structure remained intact without any catastrophic collapse. The slight volume shrinkage observed at 1300 °C is primarily attributed to densification, phase rearrangement and the elimination of residual volatile components of the aluminum phosphate binder [34]. This underscored its favorable high- and low-temperature resistance, as well as its fireproof characteristics, thereby emphasizing the application advantages of LASO NFAs in extremely challenging environments.

## 3. Materials and Methods

### 3.1. Materials

Sec-butanol (99%), aluminum sec-butoxide (95%), acetylacetone (analytical grade), and polyvinylpyrrolidone (PVP, *M_w_* = 1,300,000) were purchased from Shanghai Aladdin Reagent Co., Ltd. (Shanghai, China). Tetraethyl orthosilicate (TEOS, 99%) was obtained from Shanghai Adamas Reagent Co., Ltd. (Shanghai, China). Aluminum dihydrogen phosphate (Al(H_2_PO_4_)_3_, 37~43%) was sourced from Shijiazhuang Xinsheng Chemical Co., Ltd. (Shijiazhuang, Hebei, China).

### 3.2. Fabrication of ASO NFMs

ASO NFMs were prepared through a sol–gel electrospinning technique. Initially, a spinnable precursor solution was formulated using aluminum sec-butoxide and TEOS as the sources of aluminum and silicon (with an Al:Si molar ratio of 6:1), along with n-butanol, acetylacetonate, and deionized water serving as mixed solvents, with PVP as the polymer template. The solution underwent continuous stirring at 60 °C for 2 h in a water bath, followed by additional stirring at room temperature for 12 h. Subsequently, the precursor solution was centrifuged to separate the supernatant, which was then subjected to electrospinning. Finally, the precursor nanofiber membrane was heated at a rate of 5 °C·min^−1^ to 1000 °C in air atmosphere and held at this temperature for 1 h, yielding the ASO NFMs.

### 3.3. Preparation of Lamellar-Structured LASO NFAs

First, several ASO NFMs were cut using a circular die and stacked layer by layer to a height of 1 cm. There were then placed in a plastic Petri dish. Next, the plastic dish containing the nanofiber membranes was immersed in diluted aluminum dihydrogen phosphate aqueous solutions (different concertation of 5 wt%, 10 wt%, 20 wt%, and 30 wt%) for 1 h. Subsequently, the dish was placed in an oven for 10 h to dry after removing excess Al(H_2_PO_4_)_3_ solution; thus, uncrosslinked Al_2_O_3_-SiO_2_ nanofiber bulk materials were obtained. After that, the uncrosslinked bulk was heated at a rate of 5 °C·min^−1^ to 900 °C under air atmosphere and held at this temperature for 1 h. Under these high-temperature conditions, the Al(H_2_PO_4_)_3_ within the uncrosslinked bulk was converted to AlPO_4_, promoting interlayer bonding between the membranes. Finally, the lamellar-structured Al_2_O_3_-SiO_2_ nanofibrous aerogels (LASO NFAs) were obtained. The ceramic fibrous aerogels prepared from Al(H_2_PO_4_)_3_ concentrations of 5 wt%, 10 wt%, 20 wt%, and 30 wt% were designated as LASO-5, LASO-10, LASO-20, and LASO-30 NFAs, respectively.

### 3.4. Instruments and Characterizations

The morphological structure and elemental distribution of the ASO NFMs and relevant LASO NFAs were characterized using a field emission scanning electron microscope (FE-SEM, Gemini SEM 300, Zeiss, Oberkochen, Germany) and a scanning transmission electron microscope (TEM, Talos F200X G2, Thermo Fisher Scientific, Waltham, MA, USA). The crystal structure of the samples was analyzed with an X-ray diffractometer (Ultima IV, Rigaku Corporation, Tokyo, Japan). Elemental analysis was conducted utilizing an X-ray photoelectron spectrometer (K-Alpha+, Thermo Fisher Scientific, Waltham, MA, USA). Tensile and compressive properties were evaluated using a tensile testing machine (XQ-2, Shanghai New Fiber Instrument Co., Ltd., Shanghai, China) and a universal testing machine (XJ810, Shanghai Xiangjie Instrument Technology Co., Ltd., Shanghai, China), respectively. Flexural stiffness was assessed with a flexibility tester (RRY-1000, Hangzhou Qingtong Boke Automation Technology Co., Ltd., Hangzhou, Zhejiang, China). A custom-built bending tester was employed to subject the samples to extensive bending tests. A simultaneous thermal analyzer (STA 449F3, Netzsch, Bavaria, Germany) was utilized for thermogravimetric–differential scanning calorimetry (TG-DSC) analysis of the samples. The room-temperature thermal conductivity was determined using a thermal conductivity tester (KDRX-II; Xiangtan Xiangyi Instrument Co., Ltd., Xiangtan, Hunan, China) based on the hot-wire method. High-temperature thermal insulation performance assessments were carried out using a laboratory-designed high-temperature heating platform. Infrared images of the samples were captured via an infrared thermal imager (E95, FLIR Technologies, Wilsonville, OR, USA).

## 4. Conclusions

In summary, lamellar-structured ceramic nanofibrous aerogels were prepared by taking electrospun Al_2_O_3_-SiO_2_ nanofiber membranes as building units and employing Al(H_2_PO_4_)_3_ as a high-temperature-resistant binding agent via a 3D assembly route including layer stacking, impregnation and calcination treatments. Owing to the acceptable mechanical characteristics of ASO NFMs and the stable Al-O-Al bonding network constructed between Al_2_O_3_-SiO_2_ nanofibers, the prepared LASO-20 NFAs show steady compressive behavior. After 500 repeated loading–unloading cycles under 50% strain, its plastic deformation reaches 22.1%. Moreover, the LASO-20 NFAs with a thickness of 20 mm could effectively insulate against surface temperatures of 1000 °C down to approximate 224 °C and possess a room-temperature thermal conductivity of approximately 0.043 W·m^−1^·K^−1^, illustrating favorable high-temperature thermal insulation characteristic. Furthermore, the resulting LASO-20 NFAs possess comparatively stable performance within a wide temperature range of −196 °C to 1000 °C, which endows them with broad application prospects in thermal insulation fields.

## Data Availability

The data that support the findings of this study are available from the corresponding authors upon reasonable request.

## Supplementary Materials

The following supporting information can be downloaded at https://www.mdpi.com/article/10.3390/molecules31111934/s1: Figure S1: Tensile strength and flexural stiffness of ASO NFMs; Figure S2: Flexural stiffness of ASO NFMs after 500 reciprocating bending cycles; Figure S3: XPS spectra of LASO-20 NFAs; Figure S4: The cyclic compression curves of LASO-20 NFAs in (a) liquid nitrogen and (b) butane torch flames (five5 cycles and a compression strain of 60%). Figure S5: XRD pattern of LASO-20 NFAs after continuous calcination at 1300 °C for 24 h; Figure S6: Sintering images of LASO-20 NFAs at different temperatures; Table S1: Comparison of lamellar ceramic fiber aerogels in the literature [35,36,37,38,39,40,41,42].
