# Supplementary material for: Lamellar-Structured Al2O3-SiO2 Nanofibrous Aerogels with Favorable Compression Resilience for Efficient High-Temperature Thermal Insulation
Source: Molecules. 2026 Jun 3;31(11):1934. doi: 10.3390/molecules31111934 (PMC13258487; doi:10.3390/molecules31111934)
Supplement: Supplementary file 1 [file molecules-31-01934-s001.zip › molecules-4304999-supplementary.pdf]

molecules

Supplementary Information

Lamellar-structured Al_2O_3-SiO_2 nanofibrous aerogels with favorable compression resilience for efficient high-temperature thermal insulation

Yuxin Ma [1,†], Mengjiao Zhang [1,†], Wenqiang Wang [1], Hanwen Zhang [1], Wenzhe Li [1], Xiangxiang Gu [1], Qiuxia Fu [1,2,*], Haoru Shan [1,2,*]

[1] School of Textile and Clothing, Nantong University, Nantong 226019, China; 2315310003@stmail.ntu.edu.cn (Y.M.); 2315320005@stmail.ntu.edu.cn (M.Z.); wwq1710@163.com (W.W.); 2415310020@stmail.ntu.edu.cn (H.Z.); 2315310022@stmail.ntu.edu.cn (W.L.); 2415320003@stmail.ntu.edu.cn (X.G.); fuqx@ntu.edu.cn (Q.F.); hrshan@ntu.edu.cn (H.S.)

[2] National and Local Joint Engineering Research Center of Technical Fiber Composites for Safety and Health, Nantong University, Nantong 226019, China; fuqx@ntu.edu.cn (Q.F.); hrshan@ntu.edu.cn (H.S.)

* Correspondence: fuqx@ntu.edu.cn (Q.F.); hrshan@ntu.edu.cn (H.S.)

† These authors contributed equally to this work.

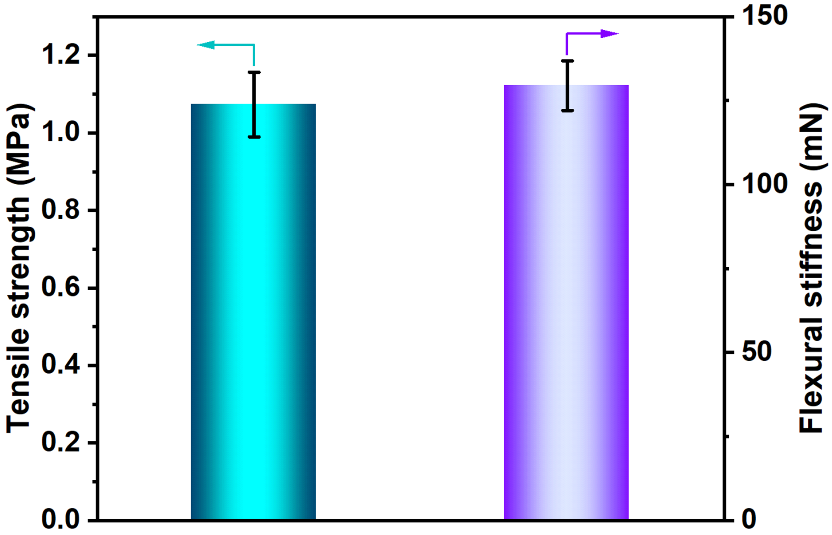

Figure S1. Tensile strength and flexural stiffness of ASO NFMs.

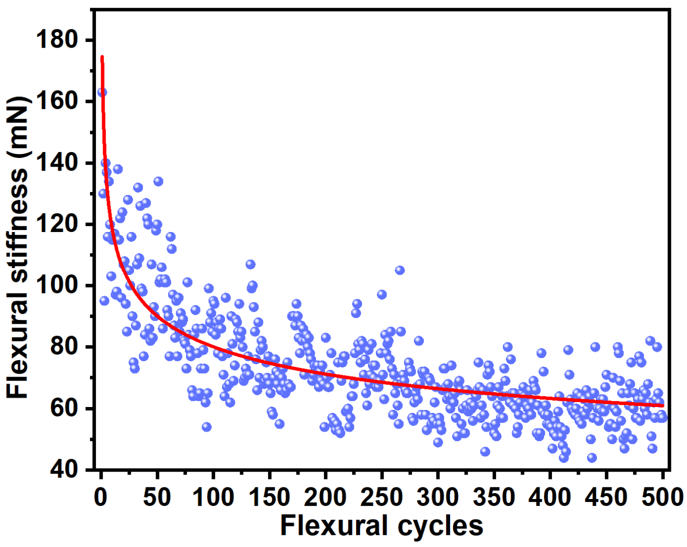

Figure S2. Flexural stiffness of ASO NFMs after 500 reciprocating bending cycles.

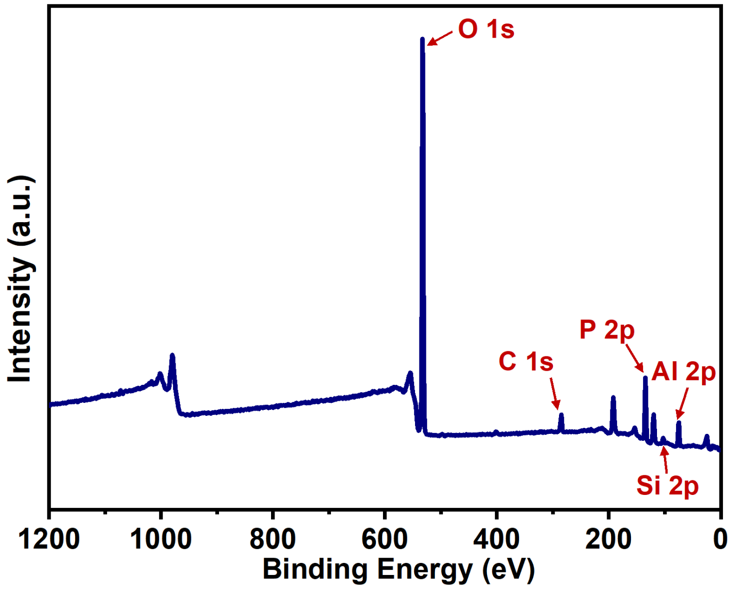

Figure S3. XPS spectra of LASO-20 NFAs.

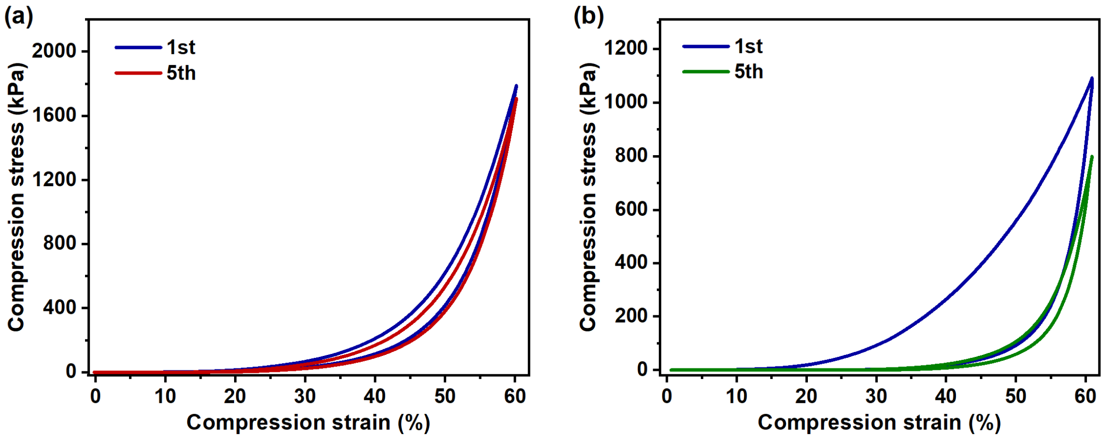

Figure S4. The cyclic compression curves of LASO-20 NFAs in (a) liquid nitrogen and (b) butane torch flames (five cycles and a compression strain of 60%).

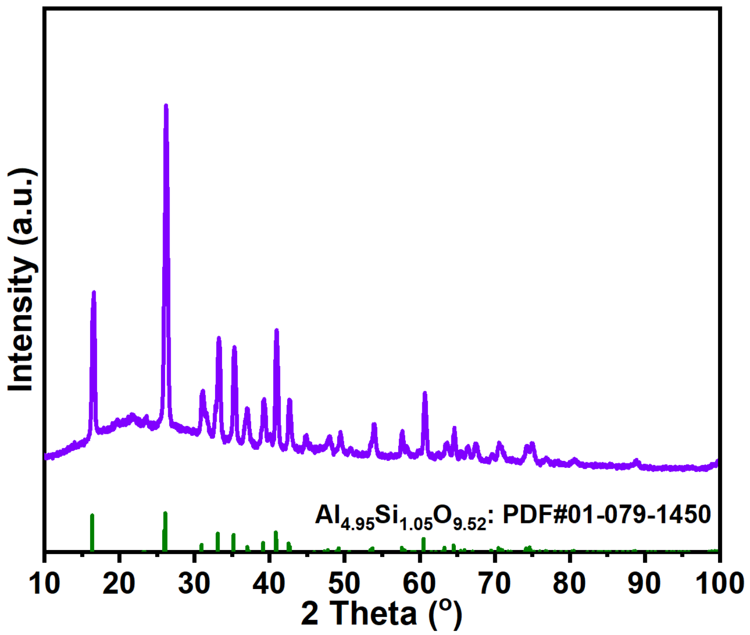

Figure S5. XRD pattern of LASO-20 NFAs after continuous calcination at 1300 °C for 24 h.

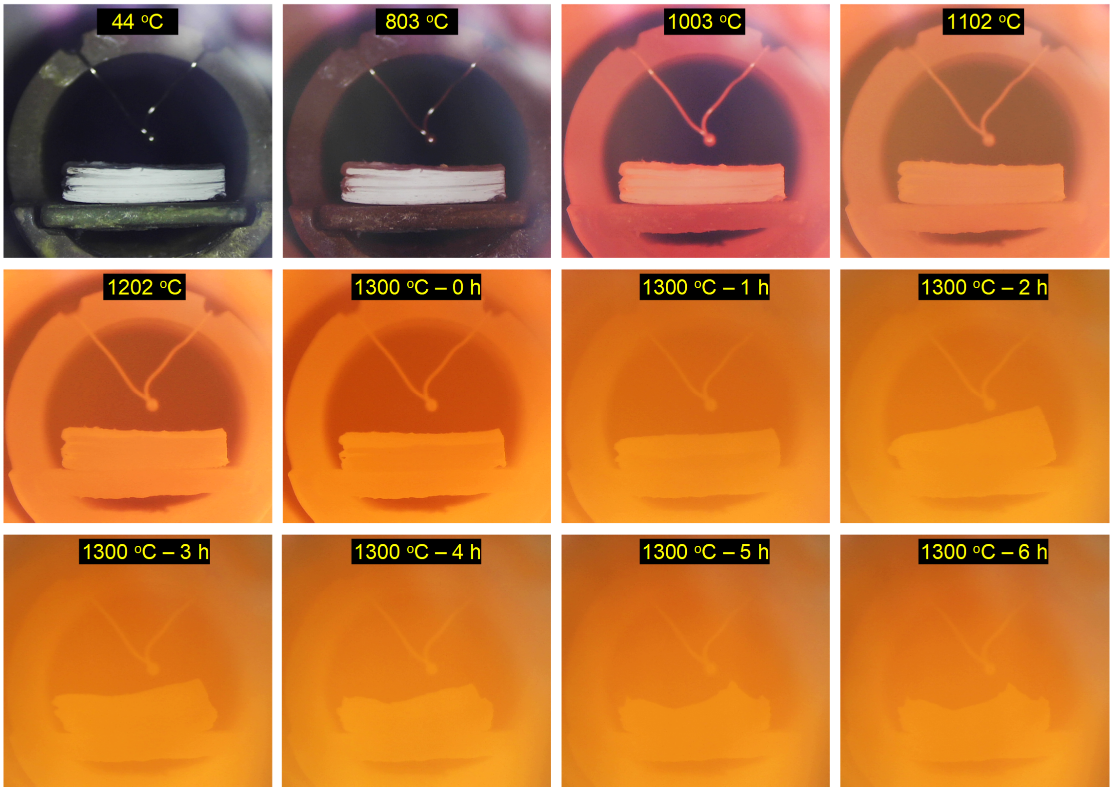

Figure S6. Sintering images of LASO-20 NFAs at different temperatures.

Table S1. Comparison of lamellar ceramic fiber aerogels in the literature.

Fiber substrate	Binding agent	Thermal conductivity ($W \cdot m^{-1} \cdot K^{-1}$)	Compressive strength (MPa)	Spatial arrangement	Compression durability	Preparation procedure	Ref.
Mullite fibers	Silicon resin	0.083 - 0.089	1.21 - 1.58	Disorderly stacking	Failure at 10 % strain	Vacuum filtration	[1]
Mullite and ZrO_2 fibers	SiC, B_4C, and starch	0.094	1.06 - 1.23	Quasi-layered structure	-	Vacuum pressing molding	[2]
Mullite fibers	SiC, B_4C, and starch	0.093 - 0.165	0.81 - 2.46	Quasi-layered structure	-	Vacuum pressing molding	[3]
Mullite and ZrO_2 fibers	SiC, B_4C, and starch	0.083-0.135	0.91 - 1.36	Quasi-layered structure	-	Vacuum pressing molding	[4]
Mullite fibers	Silica sol	0.095	1.79	Disorderly stacking	-	Vacuum filtration method	[5]
Mullite and glass fibers	Silica sol	-	2.14	Disorderly stacking	-	Vacuum filtration method	[6]
Mullite fiber	Al_2O_3 sol	0.037 - 0.217	1.03 - 5.31	Disorderly stacking	-	Vacuum filtration method	[7]
Alumina and glass fibers	Silica sol	-	0.49	Disorderly stacking	Failure at 7% strain	Vacuum filtration method	[8]
ASO NFMs	$Al(H_2PO_4)_3$	0.043	0.055 (50% strain)	Lamellar structure	500 cycles (50% strain): plastic deformation 22.1%	Face-to-face stacking	This work

References:

[1] Dong, X.; Sui, G.; Yun, Z.; Wang, M.; Guo, A.; Zhang, J.; Liu, J. Effect of temperature on the mechanical behavior of mullite fibrous ceramics with a 3D skeleton structure prepared by molding method. *Mater. Des.* **2016,** *90*, 942-948.

[2] Zhang, R.; Hou, X.; Ye, C.; Wang, B.; Fang, D. Fabrication and properties of fibrous porous mullite–zirconia fiber networks with a quasi-layered structure. *J. Eur. Ceram. Soc.* **2016,** *36*, 3539-3544.

[3] Zhang, R.; Ye, C.; Hou, X.; Li, S.; Wang, B. Microstructure and properties of lightweight fibrous porous mullite ceramics prepared by vacuum squeeze moulding technique. *Ceram. Int.* **2016,** *42*, 14843-14848.

[4] Zhang, R.; Hou, X.; Ye, C.; Wang, B. Enhanced mechanical and thermal properties of anisotropic fibrous porous mullite–zirconia composites produced using sol-gel impregnation. *J. Alloys Compd.* **2017,** *699*, 511-516.

[5] Zhu, J.; Zhu, R.; Hu, Y.; Wang, Z. Mullite fiber porous ceramic with high quality factor for high-temperature PM filtration. *J. Eur. Ceram. Soc.* **2024,** *44*, 2630-2637.

[6] Yang, Y.; Fu, W.; Chen, X.; Chen, L.; Hou, C.; Wang, Y.; Tang, T.; Zhang, X. Fabrication of homogeneous mullite-based fiber porous ceramics with high strength and porosity. *J. Eur. Ceram. Soc.* **2022,** *42*, 7219-7227.

[7] Yang, M.; Luo, X.; Yi, J.; Zhang, X.; Peng, Z. Fabrication of fibrous mullite-alumina ceramic with high strength and low thermal conductivity. *J. Wuhan Univ. Technol.* **2019,** *34*, 1415-1420.

[8] Zang, W.; Guo, F.; Liu, J.; Du, H.; Hou, F.; Guo, A. Lightweight alumina based fibrous ceramics with different high temperature binder. *Ceram. Int.* **2016,** *42*, 10310-10316.
